# Correction to: Healthcare resource utilisation and safety outcomes in patients undergoing transcatheter mitral valve repair - A nationwide analysis

**DOI:** 10.1093/eurheartj/ehae168

**Published:** 2024-03-25

This is a correction to: T Thevathasan, S Berlinghof, D Elschenbroich, J M Wiedenhofer, A L Gaul, F Barbieri, U Landmesser, M Reinthaler, C Skurk, Healthcare resource utilisation and safety outcomes in patients undergoing transcatheter mitral valve repair - A nationwide analysis, *European Heart Journal*, Volume 44, Issue Supplement_2, November 2023, ehad655.1749, https://doi.org/10.1093/eurheartj/ehad655.1749

In the originally published version of this manuscript, there was a typographical error in the second author’s last name. This should read: “S Berlinghof”.

The error has been corrected in the article.

